# Transcriptomic and epigenetic analysis of various cell types affected by amyloid‐beta oligomers in the hippocampus of mice during aging

**DOI:** 10.1002/alz70855_103920

**Published:** 2025-12-23

**Authors:** Klarissa Leduc

**Affiliations:** ^1^ Université de Montréal, Montréal, QC, Canada

## Abstract

**Background:**

Alzheimer's disease (AD) is a neurodegenerative disorder that affects memory and accounts for over 70% of dementia cases. It is characterized by the aggregation of amyloid‐beta oligomers (Aßo) and neurofibrillary tangles. Aßo accumulate 10 to 15 years before the clinical onset of AD. Several studies have shown that Aßo contribute to synaptic loss and neuronal death in the hippocampus, a brain region crucial for memory and one of the first areas affected in AD.

**Method:**

In this project, Aßo were injected daily into the hippocampus of mice aged 6, 12, and 18 months for 5 days. The hippocampus was then harvested, and single‐cell nuclei were isolated for simultaneous transcriptomic and epigenetic analysis (single nucleus RNA‐seq + ATAC‐seq; 10xGenomics). The analysis targeted neurons, astrocytes, oligodendrocytes, pericytes, endothelial cells, and microglia.

**Result:**

This study will identify gene expression changes and DNA accessibility sites induced specifically by Aßo in each cell type during aging in mice.

**Conclusion:**

A better understanding of Aß pathology could open new therapeutic avenues for preventing neurodegeneration before it causes permanent brain damage and severe memory loss, ultimately affecting cognitive health, autonomy, and quality of life in patients.

